# Structural recognition of the *MYC* promoter G-quadruplex by a quinoline derivative: insights into molecular targeting of parallel G-quadruplexes

**DOI:** 10.1093/nar/gkab330

**Published:** 2021-05-12

**Authors:** Jonathan Dickerhoff, Jixun Dai, Danzhou Yang

**Affiliations:** Purdue University, College of Pharmacy, Medicinal Chemistry and Molecular Pharmacology, 575 W Stadium Ave., West Lafayette, IN 47907, USA; College of Pharmacy, The University of Arizona, Tucson, AZ 85721, USA; Purdue University, College of Pharmacy, Medicinal Chemistry and Molecular Pharmacology, 575 W Stadium Ave., West Lafayette, IN 47907, USA; Purdue University Center for Cancer Research, 201 S University St, West Lafayette, IN 47906, USA; Purdue University, Department of Chemistry, West Lafayette, IN, USA; Purdue Institute for Drug Discovery, West Lafayette, IN, USA

## Abstract

DNA G-Quadruplexes (G4s) formed in oncogene promoters regulate transcription. The oncogene *MYC* promoter G4 (MycG4) is the most prevalent G4 in human cancers. However, the most studied MycG4 sequence bears a mutated 3′-residue crucial for ligand recognition. Here, we report a new drug-like small molecule PEQ without a large aromatic moiety that specifically binds MycG4. We determined the NMR solution structures of the wild-type MycG4 and its 2:1 PEQ complex, as well as the structure of the 2:1 PEQ complex of the widely used mutant MycG4. Comparison of the two complex structures demonstrates specific molecular recognition of MycG4 and shows the clear effect of the critical 3′-mutation on the drug binding interface. We performed a systematic analysis of the four available complex structures involving the same mutant MycG4, which can be considered a model system for parallel G4s, and revealed for the first time that the flexible flanking residues are recruited in a conserved and sequence-specific way, as well as unused potential for selective ligand-G4 hydrogen-bond interactions. Our results provide the true molecular basis for MycG4-targeting drugs and new critical insights into future rational design of drugs targeting MycG4 and parallel G4s that are prevalent in promoter and RNA G4s.

## INTRODUCTION

Cancer is now the second leading cause of death globally and the most common cause in high-income countries (1). The *MYC* oncogene is a central player in the majority of human tumors (2–6) and is a general amplifier of transcription in cancers (7). However, the MYC protein is often considered ‘undruggable’ (8). DNA G-quadruplexes (G4s) are found to form in the promoter regions of human oncogenes (9). The G4 formed in the nuclease hypersensitive element (NHE III_1_) within the proximal promoter region of the *MYC* oncogene functions as transcriptional silencer (10). Small molecules can bind to the *MYC* promoter G4 to down-regulate *MYC* transcription and induce cancer cell death (11,12). G4 formation is elevated in human precancerous cells and G4 structures are enriched in the *MYC* promoter of highly transcribed cells (13). Therefore, the *MYC* G4 is considered a promising target for cancer therapeutics (9,10,14). However, few G4-binding molecules have drug-like properties critical for drug development purpose (15).

G-quadruplexes are noncanonical four-stranded DNA secondary structures formed from guanosine-rich sequences. Four guanines constitute a cyclic G-tetrad held together by Hoogsteen hydrogen bonds. Physiologically abundant monovalent cations, mostly potassium and sodium ions, facilitate the stacking of usually three tetrads into a G4. G4s are of globular shape and exhibit large structural diversity with different folding and capping conformations, thus offering unique opportunities for selective targeting by small molecules (16,17).

**Figure 1.**
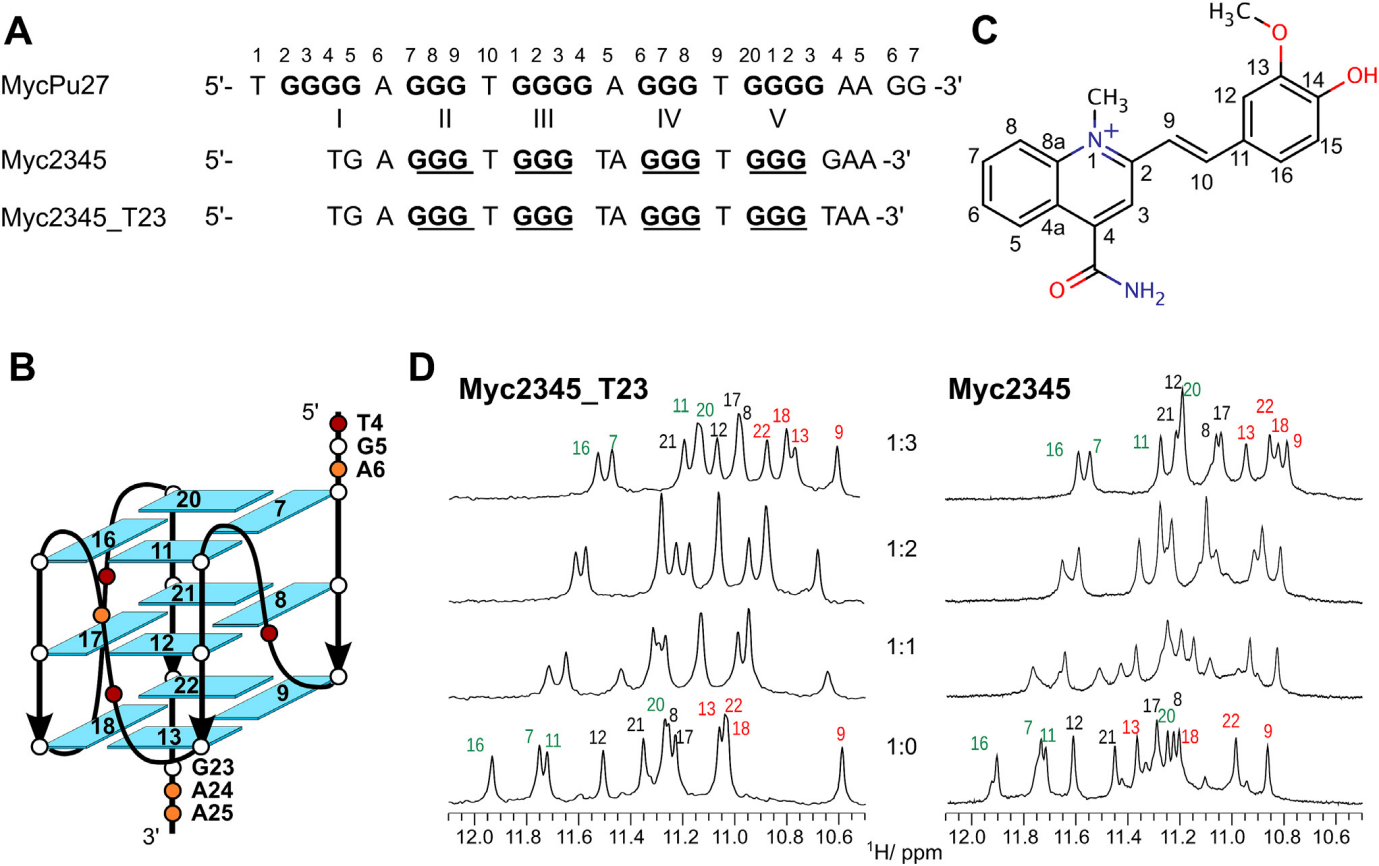
(**A**) Sequence of the MycPu27 motif and its derivatives. (**B**) Schematic structure of the Myc2345 G4. (**C**) Structure of the quinoline derivative PEQ. (**D**) 1D NMR spectra showing the imino region of Myc2345_T23 and Myc2345 during the titration with PEQ. The imino protons of the 5′-, central, and 3′-tetrad are labeled in green, black, and red, respectively. Recorded with 10 mM K^+^, pH 7 at 25°C.

The G-rich NHE III_1_ (MycPu27, Figure 1A) in the *MYC* promoter is one of the most studied G4 forming sequences (10). The major G4 structure is a parallel structure with a 1:2:1 nt loop arrangement that involves G-tracts 2–5 in the G-core with G14 and G23 not involved in the tetrad formation (Figure 1B) (11,18). This major MYC G4 is represented by the structure of Myc2345_T23 that was previously determined by NMR in 2005 (18). The Myc2345_T23 sequence bears a G23-to-T mutation in the 3′-flanking at position 23, in addition to the G14-to-T mutation in the second propeller loop (Figure 1A). This sequence is one of the most studied G4s and serves as the model structure for the important type of parallel G4s with short propeller loops that is over-represented in the human genome (19,20). However, using large-scale G4 DNA microarrays with ten-thousands of different G4s, the position 23 in the 3′-flanking was very recently shown to play a critical role in selective ligand recognition of MycG4 and parallel G4s even in the context of extended DNA sequences (21).

In this study, we first determined the NMR solution structure of the Myc2345 sequence, which contains the wild-type G23 in the 3′-overhang, a key residue for ligand recognition and selectivity. In the context of ligand recognition, we refer to this sequence as the wild-type Myc2345 because the G14-to-T mutation in the short second propeller loop is not involved in the ligand interactions. We discovered a new small molecule PEQ (phenyl-ethenyl-quinoline, NSC85697) (Figure 1C) that has good drug-like properties and specifically binds *MYC* G4s. We determined the high-resolution NMR solution structures of the 2:1 complexes of PEQ with the wild-type Myc2345 and mutant Myc2345_T23.

Comparison of the two structures shows a notable effect of this critical mutation on the drug binding interface. Moreover, the recognition of Myc2345_T23 has now been described for four small molecules by high-resolution NMR structures including the newly resolved PEQ complex (15,22,23). A systematic analysis of the four available complex structures revealed that for recognition of parallel G4s the flexible flanking residue is recruited in a conserved way specific to the flanking sequence. This newly uncovered feature of parallel G4 recognition by small molecules can enable rational drug design as well as in-situ drug screening using our determined structure. Therefore, our results provide novel and critical insights into future rational design of small molecules targeting MycG4 and parallel G4s for cancer therapeutics.

## MATERIALS AND METHODS

### Sample preparation

The NSC85697 (PEQ) was obtained from the National Cancer Institute (NCI) and solved in d_6_-DMSO or DMSO for NMR or optical experiments, respectively. Unmodified oligonucleotides were synthesized and purified as described previously (24). The 3′-TAMRA labeled Myc2345_T23 sequence was purchased from Sigma-Aldrich. The DNA samples were annealed by heating to 95°C for 5 min and subsequent slow cooling to room temperature. The DNA concentration was quantified based on the UV/vis absorption at 260 nm.

### Circular dichroism (CD) spectroscopy

All CD experiments were performed using a JASCO-1100 spectropolarimeter (JASCO, Inc.) equipped with a temperature controller. CD spectra of 5 μM DNA samples in 25 mM potassium phosphate buffer, 75 mM KCl, pH 7 were acquired at 25°C with and without PEQ at 5:1 ligand:DNA ratio. A 10 mm quartz cuvette was used and the spectra were recorded with three accumulations, a data pitch of 1 nm, a 1 mm band width, a response time of 2 s, and a scanning speed of 50 nm/min. All spectra were blank corrected by subtracting the buffer spectrum.

For CD melting experiments, 5 μM DNA samples were prepared in 0.6 mM potassium phosphate buffer with and without PEQ at 5:1 ligand:DNA ratio. A 10 mm quartz cuvette was used and ellipticity at 260 nm was recorded between 25 and 95°C with a heating rate of 1°C/min, a data point every 0.5°C, a bandwidth of 1 nm, and a digital integration time of 2 s. The melting temperature was determined at the intersection of the melting curve with the median of the fitted baselines. Every melting temperature was measured in duplicate.

### Fluorescence spectroscopy

All fluorescence spectra were acquired on a Jasco FP-8300 spectrofluorometer (JASCO, Inc.) equipped with a temperature controller at 25°C. A 100 nM sample of 3′-TAMRA-labeled Myc2345_T23 was prepared in 25 mM potassium phosphate buffer, 75 mM KCl, pH 7. The emission spectrum between 565 and 610 nm was recorded with an excitation wavelength of 555 nm, excitation and emission bandwidth of 5 nm, a response time of 4 s, a data interval of 1 nm, and a scanning speed of 50 nm/min. An initial titration of PEQ to the 3′-TAMRA-labeled Myc2345_T23 was performed to determine the optimal ligand-DNA ratio for the competition experiments. A 10:1 ratio was chosen to obtain significant TAMRA quenching while limiting the excess of PEQ in solution. The emission at 579 nm was used to follow the changes in TAMRA fluorescence upon ligand or DNA addition. Myc2345 or Myc2345_T23 were titrated to the 10:1 mixture of PEQ and 3′-TAMRA labeled Myc2345_T23 in duplicate to determine the *C*_50_ value, the concentration of the competing unlabeled DNA sequence at which half the initially quenched fluorescence is restored. For this purpose, the normalized change in TAMRA fluorescence Δ*F* was fitted against Δ*F*_max_/(1 + (*C*_50_/*x*)*^n^*) with Δ*F*_max_ as maximum change in fluorescence, *x* as the concentration of the competing DNA sequence, and n as slope.

### Nuclear magnetic resonance (NMR) spectroscopy

DNA for NMR samples was solved in 10 mM potassium phosphate buffer with 90% H_2_O/10% D_2_O. The 1.4 mM Myc2345 and 0.8 mM Myc2345 + PEQ samples were measured at pH 7.0 on a Bruker AV-800 spectrometer with a cryoprobe and the 0.7 mM Myc2345_T23 + PEQ sample was measured at pH 6.5 on a Bruker DRX-600 spectrometer. The temperatures were between 15°C and 40°C and excitation sculpting was employed for water suppression. The NOESY mixing times were between 80 ms and 300 ms. Additional ^1^H–^13^C HSQC and DQF-COSY experiments were performed for the Myc2345 and Myc2345 + PEQ sample with an HSQC optimized for a ^1^*J*(C,H) of 190 Hz. Chemical shift referencing was done directly for ^1^H based on the water signal relative to DSS and indirectly for ^13^C relative to DSS. The spectra were processed with Topspin 3.5 (Bruker) and analyzed with CcpNmr Analysis 2.4 (25).

### Structure calculation

The high-resolution structures were calculated based on the NMR data as described previously (26) using Xplor-NIH (27,28) and the Amber 16 package (29). Some modification and additional steps were necessary for the determination of the G4-ligand structures: The PEQ ligand was parameterized for the Amber force field using the R.E.D. server (30). Instead of a distance geometry simulate annealing (DGSA) approach to create the starting structures for the DNA-ligand complexes, an Xplor-NIH simulated annealing protocol was used starting at a temperature of 10 000 K. In this way, diverse starting structures were independently created from an extended DNA conformation with unbound ligands, instead of the possibly biased manual creation of a single G4-ligand model as starting structure. Broader boundaries of ±1.5 Å and ±2 Å were used for intermolecular restraints between ligand and DNA involving exchangeable and non-exchangeable protons, respectively.

## RESULTS

### PEQ strongly binds the MYC promoter G4

We discovered a quinoline derivative PEQ (phenyl-ethenyl-quinoline, NSC85697) (Figure 1C) as a specific *MYC* G4 binder by screening of the National Cancer Institute (NCI) 2K chemical diversity library and a subsequent compound homology search (12). The new PEQ compound has good drug-like properties that are crucial for drug development (15,31). Extended aromatic ring systems are characteristic for most G4-ligands to maximize stacking interactions with the external G-tetrad. Unlike most other G4-ligands, the PEQ compound consist of a phenyl and quinoline moiety linked via a more flexible ethenyl bridge instead of a large fused aromatic ring-system. The methylated N1 of PEQ carries a positive charge of +1.

We first tested the PEQ binding to Myc2345_T23 and the wild-type Myc2345 using CD experiments. Myc2345 contains the wild-type G23 in the 3′-overhang, whereas Myc2345_T23 contains the G-to T mutation T23 to prevent a 1:2:2 loop isomer that utilizes G23 for the G-core (Figure 1A). The same parallel folding of Myc2345 and Myc2345_T23 is suggested by their similar CD spectra with a large positive band at about 260 nm and a negative band at 240 nm, which is characteristic of a parallel G-core with homopolar stacked tetrads (Supplementary Figure S1) (32).

Ligand addition to the G4s only slightly changed the CD spectra compared to the free form, however, the thermal stability of the PEQ-G4 complex was increased by about 20°C for both sequences (Supplementary Figure S1 and Supplementary Table S1). Therefore, PEQ binds and stabilizes the wild-type Myc2345 and Myc2345_T23 without perturbation of their parallel topology. 1D ^1^H NMR titrations were used to investigate the PEQ binding. Upon PEQ addition, most DNA imino proton resonances of both Myc2345_T23 and Myc2345 shift up-field, indicating binding of one molecule to each external tetrad via end-stacking (Figure 1D). The promising spectral quality suggested that determination of the high-resolution structures of both ligand-G4 complexes was feasible.

### Solution structure of the wild-type Myc2345

We first determined the high-resolution structure of Myc2345 G4 with the wild-type G23 by solution NMR. The Myc2345 sequence formed one major species as shown by the 12 predominant imino proton resonances in the 1D ^1^H NMR spectrum (Figure 1D). Only a slightly populated minor species is visible, likely corresponding to the 1:2:2 loop isomer utilizing G23 in the G-core (33). The same folding topology of Myc2345 and Myc2345_T23 is supported by the strong resemblance of the 5′-tetrad imino proton resonances in the 1D NMR spectra of both sequences, which contain the identical 5′-flanking (Figure 1D), and comparable melting temperatures of 57.5 and 54.6°C in the presence of 0.6 mM potassium ions for Myc2345 and Myc2345_T23, respectively (Supplementary Table S1). 2D NMR spectra, including NOESY, HSQC and DQF-COSY experiments, were collected and assigned (Supplementary Figure S2 and Supplementary Table S2). All residues adopt *anti* glycosidic torsion angles based on the H6/H8-H1′ NOE cross peak intensities and the C6/C8 carbon chemical shifts. In addition, most residues could be assigned to a *south* sugar pucker conformation based on the stronger H1′-H2′ cross peaks compared to the corresponding H1′-H2″ peaks in the DQF-COSY spectrum (Supplementary Figure S2D).

**Figure 2.**
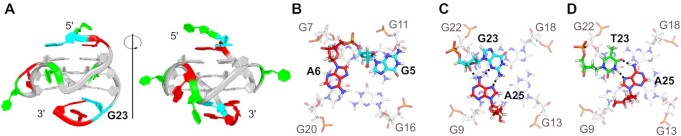
(**A**) Structure of the Myc2345 G4 (PDB: 7KBV). (**B**) 5′- and (**C**) 3′-capping of Myc2345. **(D)** 3′-capping of Myc2345_T23. Hydrogen bonds are indicated by black dashed lines.

The high-resolution structure of Myc2345 was calculated via restrained molecular dynamics based on distance information extracted from the NOESY experiments at various temperatures and mixing times (Supplementary Tables S3 and S4) as described previously (26). A well-converged ensemble of the 10 lowest-energy structures was obtained with a heavy atom root-mean-square deviation (RMSD) of 0.47 Å for the G-core and 0.76 Å for all residues (Table 1, Figure 2 and Supplementary Figure S3A). Within the determined parallel Myc2345 G4, all thymidine bases of the three propeller loops are solvent exposed. Only the A15 in the central loop points into the groove as shown by a A15-H2/G17-H8 NOE cross-peak. The 5′-tetrad is covered by G5 and A6 (Figure 2A and B), whose Watson–Crick edges point towards opposite grooves. Several NOE contacts to tetrad H1 imino protons locate the flanking G5 above G11, such as G5–H1′ to G7–H1 and G11–H1 or G5–H8 to G11–H1 and G16–H1 (Supplementary Table S3). The position of A6 above G20 and G7 is defined by A6-H1′/G20-H1 and A6–H2/G20-H1′ NOEs as well as those between G20-H1 and A6 aromatic protons H2 and H8. The 5′-capping is concluded by the terminal T4 that stacks on A6 (Figure 2A) as defined by a T4-Me/A6-H2 cross peak and NOEs of the T4 sugar protons with A6-H8 and G5-H8 (Supplementary Table S3).

**Table 1.** NMR restraints and structural statistics

	Myc2345	Myc2345 +PEQ	Myc2345_T23+PEQ
**NOE-based distance restraints**			
Intra-residue	286	354	341
Inter-residue			
Sequential	104	136	139
Long-range	51	31	30
PEQ-G4	-	42	41
**Other restraints**			
Hydrogen bonds	48	48	48
Torsion angles	40	39	22
G-tetrad planarity	36	36	36
**Structural statistics**			
**Pairwise heavy atom RMSD (Å)**			
G-tetrad core	0.47 ± 0.13	0.33 ± 0.08	0.63 ± 0.17
All residues	0.76 ± 0.15	0.87 ± 0.35	1.01 ± 0.36
**Restraint violations (Å)**			
Mean NOE	0.001 ± 0.007	0.001 ± 0.008	0.001 ± 0.007
Max. NOE	0.17	0.20	0.14
**Deviations from idealized geometry**			
Bonds (Å)	0.01 ± 0.00	0.01 ± 0.00	0.01 ± 0.00
Angles (°)	2.15 ± 0.02	2.25 ± 0.02	2.29 ± 0.04

On the 3′-end, the terminal A25 folds back and forms an internal G23-A25 base pair that covers the 3′-tetrad (Figure 2C). Although the formation of a T23–A25 base pair also occurs for the Myc2345_T23 sequence, the capping structures differ in the pairing and the orientation of residue A25 (Figure 2C and D). Whereas the T23–A25 Hoogsteen base pair centrally stacks on the 3′-tetrad in Myc2345_T23 with A25-H2 pointing towards the G13-G18 edge, a reversed orientation of A25 is found for Myc2345 with its H2 located above the G9–G22 edge. Consequently, the Hoogsteen edge of A25 interacts with the sugar edge of G23 via three potential hydrogen bonds and the base pair covers G9 and G22. This conformation is supported by numerous NOE cross peaks between the 3′-flanking and the external 3′-tetrad (Supplementary Table S4). Several sequential contacts including an H8-H8 interaction confirm the stacking of G22 and G23, while A25’s position is well defined by many NOE interactions with G9 such as A25–H2 to G9–H1/H8/H2′/H2″, A25–H8/G9–H1 and A25–H1′/G9–H1.

Chemical shift differences between the G-core H1 and H8 protons of the two sequences also reflect the different position of the 3′-capping structure (Supplementary Figure S3B). In Myc2345, the stacking of the G23-A25 pair upon G9 and G22 induces a shielding of G9-H8 and deshielding of the G9 and G13 H1 protons (Figure 2C).

### NMR structure determination of the 2:1 complexes of PEQ with the Myc2345 and Myc2345_T23 G4

**Figure 3.**
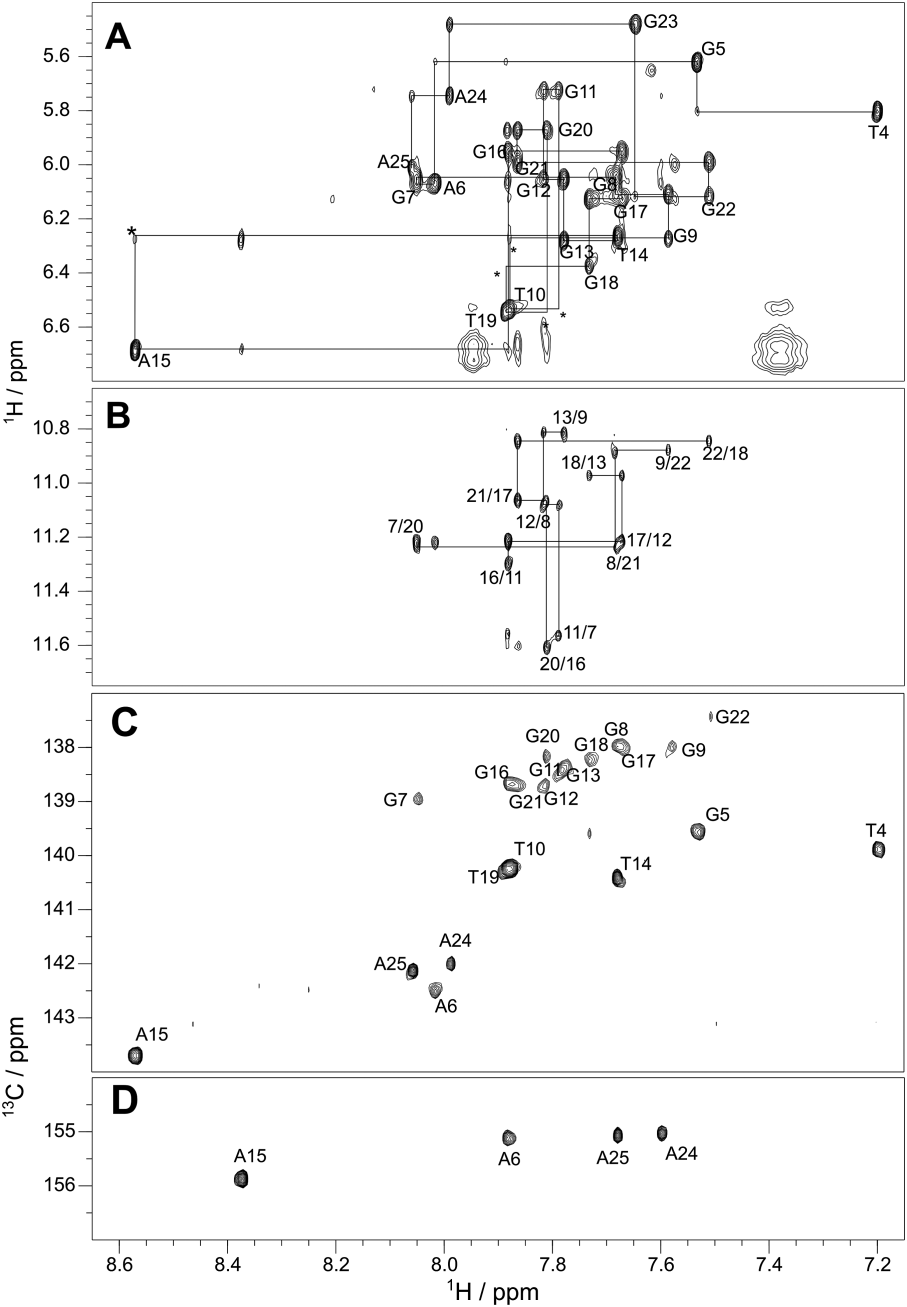
2D NOE spectral regions of the 2:1 PEQ-Myc2345 complex. (**A**) Sequential H6-H1′ and H8-H1′ contacts traced by solid lines. Missing NOE cross peaks are marked with an asterisk. (**B**) Guanine H8–imino cross peaks showing the intra-tetrad and inter-tetrad guanine connectivities indicated by solid lines. Spectral regions of the 2D ^1^H−^13^C HSQC of the 2:1 PEQ-Myc2345 complex showing the (**C**) H6−C6/H8−C8 peaks for all bases and the (**D**) adenine H2−C2 contacts. All spectra acquired at 25°C in 10 mM K^+^ buffer, pH 7. NOESY mixing time is 300 ms.

**Figure 4.**
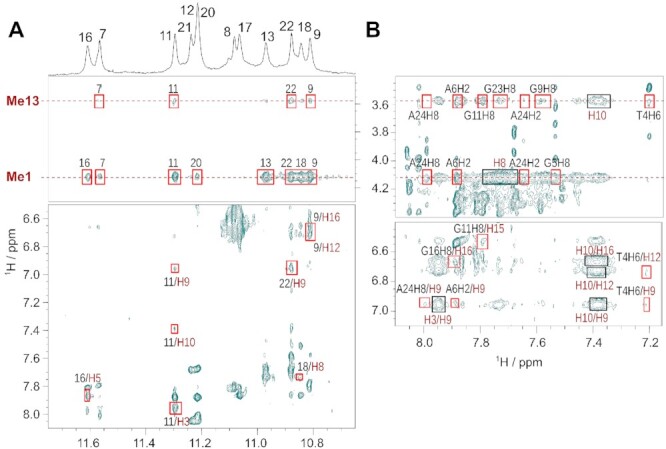
2D NOESY spectral regions showing the intermolecular NOE contacts (red) between (**A**) PEQ and G-core imino protons and (**B**) PEQ and H2/H6/H8 aromatic protons. PEQ protons are labeled in red. Strong intramolecular NOE cross peaks of PEQ are indicated by a black box. Spectrum measured at 25°C with 10 mM K^+^, pH 7 and 3:1 ligand-DNA ratio. NOESY mixing time is 300 ms.

Various 2D NMR spectra were recorded for the PEQ complexes of Myc2345 and Myc2345_T23 at 3:1 PEQ-DNA ratio including NOESY, HSQC, and DQF-COSY experiments (Figures 3, 4, Supplementary Figures S4 and S5) with complete assignments (Supplementary Tables S5–S8). A total of 521 and 510 intra-molecular NOESY cross-peaks were identified for Myc2345 and Myc2345_T23 in complex with PEQ, respectively, including inter-residue and intra-residue interactions (Table 1 and Supplementary Tables S9 and S10). In addition, over 40 inter-molecular interactions between G4 and PEQ were identified for each complex (Table 1 and Supplementary Tables S11 and S12, Figure 4 and Supplementary Figure S5D, E). They are almost equally distributed among the two binding sites and in line with end-stacking of one PEQ molecule to each the 5′- and 3′-tetrad.

The two methyl groups Me1 and Me13 of PEQ (Figure 1C) at about 4.1 and 3.6 ppm, respectively, give rise to many clearly assigned inter-molecular cross-peaks, because their signals are in a region free of DNA resonances (as detailed below).

### NMR solution structure of the 2:1 complex of PEQ with the Myc2345 G4

**Figure 5.**
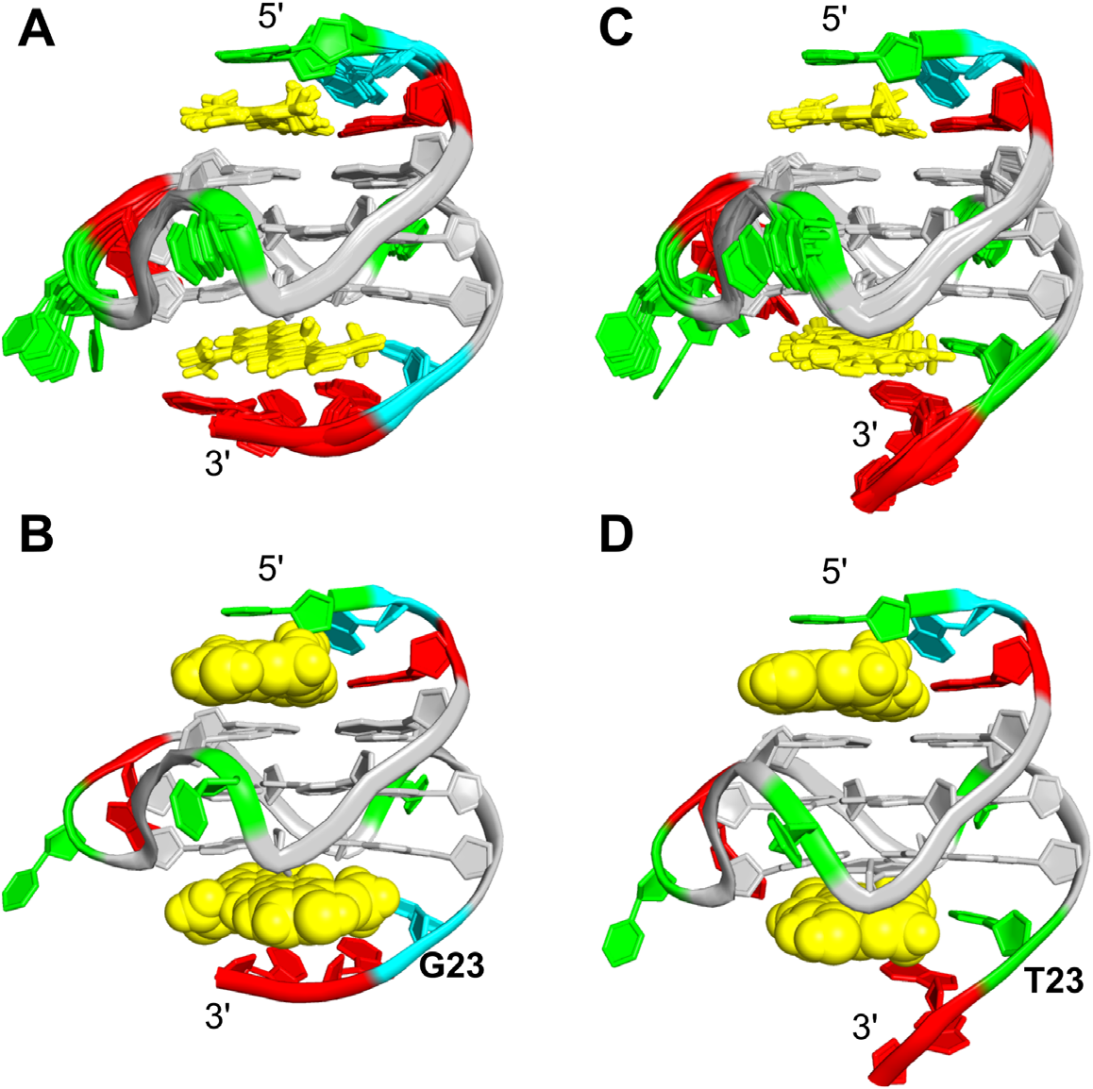
(A, B) Superposition of the 10 lowest energy structures (**A**) and a representative NMR structure (**B**) of the 2:1 PEQ complexes with Myc2345 (PDB: 7KBW). (C, D) Superposition of the 10 lowest energy structures (**C**) and a representative NMR structure (**D**) of the 2:1 PEQ complexes with Myc2345_T23 (PDB: 7KBX).

Based on the NOE distance restraints, the high-resolution structure of the 2:1 complex of PEQ with Myc2345 was determined as described for the free G4. The 10 lowest energy structures are well-converged for the complex (Table 1, Figure 5A).

**Figure 6.**
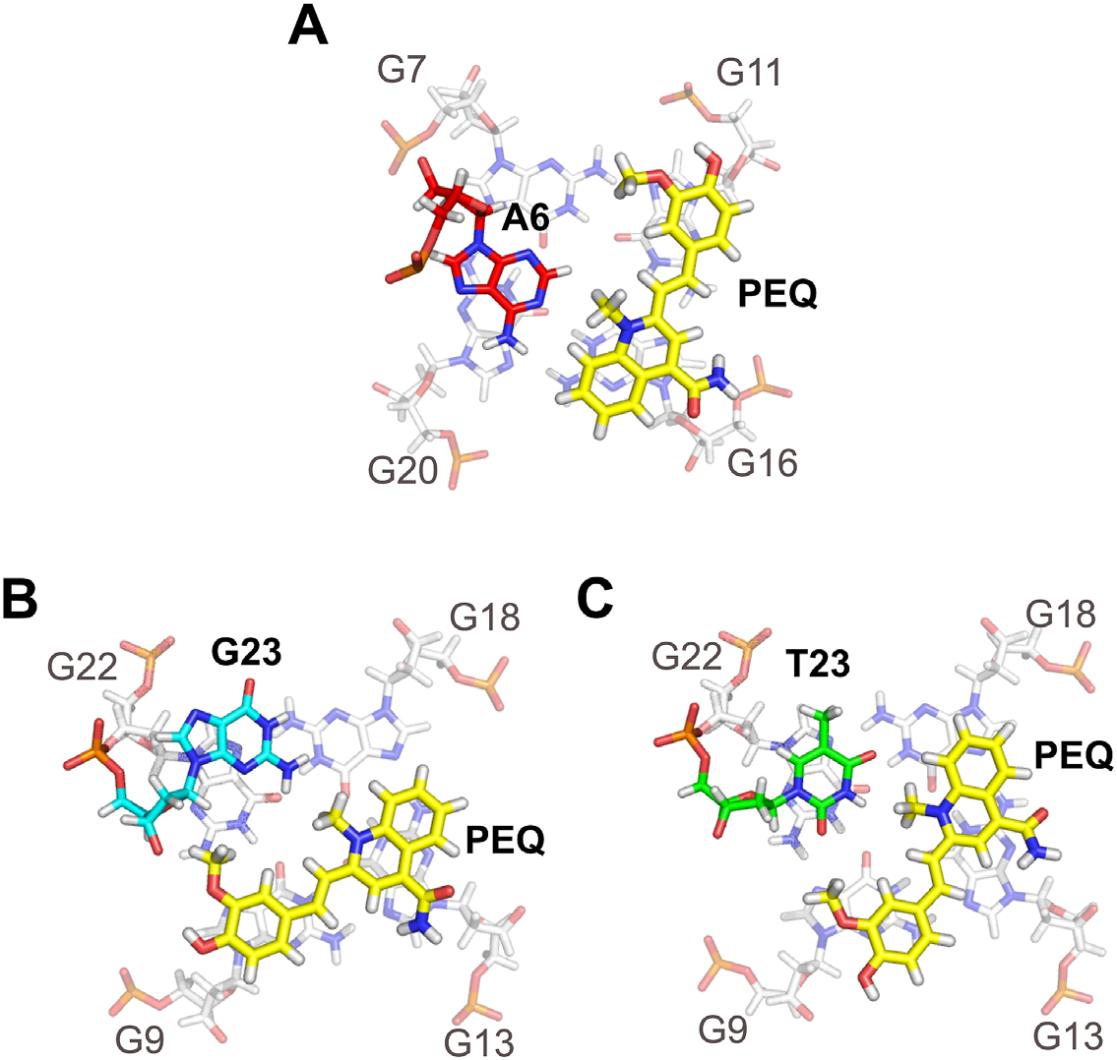
Structural details of the PEQ recognition. (**A**) 5′-site and (**B**) 3′-site of Myc2345 in the 2:1 PEQ complex. (**C**) 3′-site of Myc2345_T23 in the PEQ complex.

In the structures of the 2:1 PEQ complex with Myc2345, the parallel G-cores and the three 1:2:1 nt propeller loops remain unchanged, whereas the 5′- and 3′-flanking sequences rearrange to form binding pockets for PEQ (Figure 5B). At the 5′-end, PEQ recruits A6 to form a ligand-base plane that maximizes the stacking interactions with the 5′-tetrad (Figure 6A). Compared to the free G4 (Figure 2B), the A6 residue is flipped and now follows the right-handed backbone of the G-core, whereas its Watson-Crick edge points towards the tetrad center. Thus, PEQ faces the Watson–Crick edge of A6 as shown by NOE cross-peaks of A6-H2 to the G7 and G20 imino protons as well as to the Me1, Me13 and H9 protons of PEQ (Supplementary Tables S9 and S11).

The phenyl and quinoline moieties of PEQ completely cover G11 and G16, respectively, with both methyl groups pointing towards A6 (Figure 6A). More precisely, the methyl group Me1 attached to the positively charged N1 of the quinoline is close to the negatively polarized central pore of the G4 as corroborated by medium NOE cross peaks to all imino protons of the 5′-tetrad, whereas the Me13 on the phenyl moiety only interacts with the G7 and G11 imino protons (Supplementary Table S11). Additionally, a PEQ-H15/G11-H8 cross-peak locates the phenyl group above G11. Moreover, the ethenyl bridge connecting the phenyl and quinoline moieties is positioned over the G11-G16 interface as shown by NOE cross-peaks between G11-H1 and the PEQ-H3, H9 and H10 resonances. The PEQ-A6 layer separates the first two residues T4 and G5 from the G-core and prevents any NOE interactions with the 5′-tetrad in contrast to the free Myc2345 (Supplementary Tables S3 and S9). Instead, G5 partially covers the quinoline moiety as shown by a G5-H8/Me1 NOE cross peak and T4 stacks over the phenyl moiety of PEQ, which is supported by several ligand NOE interactions with T4-H6 and T4-Me (Supplementary Figures S5E, S6A, and Supplementary Table S11).

At the 3′-end, the 3′-flanking residue G23 is specifically recruited for the ligand-base plane that stacks over the 3′-external tetrad (Figure 5A and B). Inter-molecular contacts that define the 3′-binding sites are summarized in Supplementary Tables S11 and S12. Like the 5′-binding, the side of PEQ with two methyl groups faces the recruited residues G23 (Figure 6B). PEQ-Me1 of the quinoline moiety is positioned centrally above the 3′-tetrad as indicated by medium NOE contacts to the four corresponding imino protons. Additional cross-peaks between PEQ-Me13 of the phenyl moiety and G9-H1/H8 and G22 H1 specify the location of the phenyl ring above G9, whereas the quinoline moiety mainly covers G13 as supported by G18-H1/PEQ-H8 and G22-H1/PEQ-H9 interactions. The PEQ-G23 plane is capped by A24 and A25 (Supplementary Figure S6B/C). A24 stacks on PEQ as shown by a A24–H2/PEQ–H8 NOE interactions, whereas A25 stacks on A24 following the right-handedness of the G-core.

Interestingly, a strong resemblance between the free and complex form is observed for the residue 23 according to the similar pattern of sequential NOE interactions, where G23 stacks on the five-membered ring of G22 (compare Figures 2C and 6B). The PEQ displaces A25 in the free Myc2345 as binding partner of the G23 base and interacts analogously with its sugar edge.

### NMR solution structure of the 2:1 complex of PEQ with Myc2345_T23 shows a notably different 3′-binding

The 3′-flanking residue 23 is recruited for ligand binding and has been shown to play a key role for binding selectivity of MYC G4 and parallel G4s (21). To understand the molecular basis of this critical mutation for ligand binding selectivity, we also determined the structure of the 2:1 PEQ complex with Myc2345_T23, which bears the G-to-T mutation at position 23 in the 3′-overhang (Table 1). The 10 well-converged lowest energy structures are shown in Figure 5C. Both Myc2345 and Myc2345_T23 share the same 5′-flanking sequence and therefore the 5′-recognition of PEQ is identical for the two complexes (Figure 6A). In contrast to the 5′-end, the 3′-binding of PEQ markedly differs for Myc2345_T23 and Myc2345 (Figure 6B and C). Inter-molecular contacts that define the 3′-binding sites are summarized in Supplementary Tables S11 and S12. PEQ recruits the mutated 3′-flanking residue T23 to form a ligand-base plane stacking on the 3′-external tetrad. Comparison of the solution structures of PEQ in complex with the wild-type Myc2345 and mutant Myc2345_T23 clearly shows the effect of this critical mutation on the drug binding interface (Figure 6B and C). The point mutation of the recruited nucleotide at position 23 notably affects the PEQ interactions because of the different size, shape, and composition of thymine and guanine. In contrast to interacting with the sugar edge of the wild-type G23, PEQ interacts with the Watson-Crick edge of the mutated T23, which stacks over the six-membered ring of G22 instead of its five-membered ring. Compared to the wild type Myc2345 complex, PEQ is rotated by about 30° along the central G4 axis in the Myc2345_T23 complex and partially covers G18. This different orientation in turn controls the position of PEQ’s functional groups. For example, whereas the carboxamide group on the quinoline ring is clearly positioned in the G13/G18 groove for Myc2345_T23, it is shifted towards the G9/G13 groove in the Myc2345 complex (Figure 6B and C). In addition, the hydroxyl and methoxy groups of the phenyl ring flank the G9 sugar in Myc2345_T23 but are both located in the G9/G22 groove for Myc2345. This result suggests that those positions of PEQ are potential sites for rational modification to optimize specific interactions with different target G4s. Noteworthy, Me13 shows NOE cross-peaks to all imino protons of the 3′-tetrad at high threshold levels for both complexes, although it is positioned above the G9/G22 edge (Figure 4A and Supplementary Figure S5D). Those peaks cannot be explained by a single binding event and indicate a slightly populated second binding mode at the 3′-end.

### Relative binding affinity of PEQ to Myc2345 and Myc2345_T23

We examined the relative binding affinity of PEQ to the wild-type MYC2345 and mutant Myc2345_T23, using a fluorescence-based competition assay (34). For this purpose, a TAMRA-fluorophore was attached to the 3′-terminus of Myc2345_T23 to create a labeled sequence, whose fluorescence is quenched upon PEQ binding (Supplementary Figure S7A). Titration of unlabeled Myc2345 or Myc2345_T23 DNA to this solution restores the TAMRA fluorescence because PEQ is depleted from the labeled G4. Based on the obtained C_50_ values of 3.71 μM for Myc2345_T23 and 4.86 μM for Myc2345, the concentration at which 50% of the initial fluorescence is restored, the binding affinity of PEQ to the different sequences is comparable (Supplementary Figures S7B and C).

### Systematic analysis of the molecular recognition of MYC2345_T23 by small molecules

**Figure 7.**
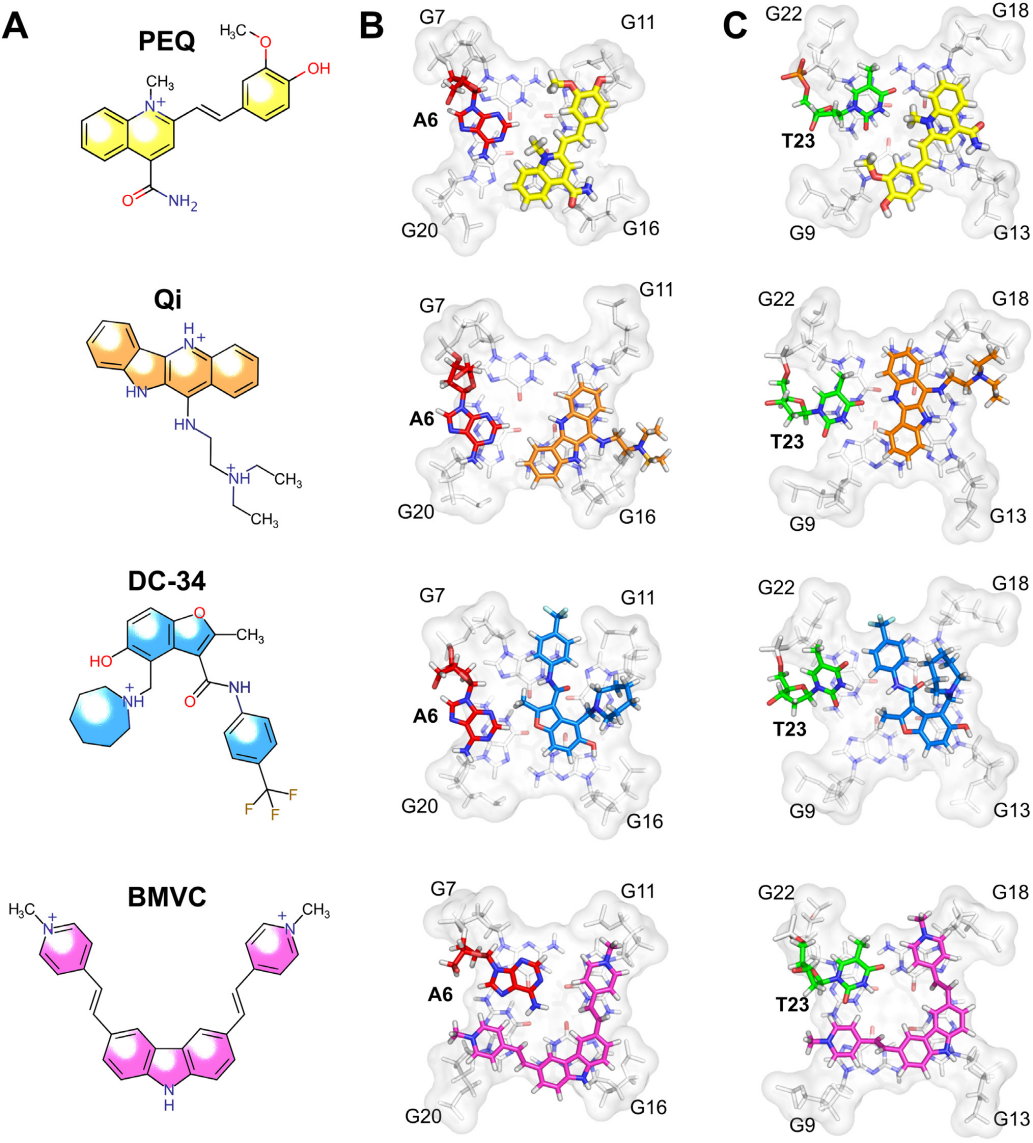
Comparison of high-resolution structures of Myc2345_T23 in complex with PEQ (PDB: 7KBX), Quindoline-i (PDB: 2L7V), DC-34 (PDB: 5W77) and BMVC (PDB: 6O2L). (**A**) Chemical structures of the ligands. (**B**) 5′-sites and (**C**) 3′-sites of ligand-Myc2345_T23 complexes.

The Myc2345_T23 sequence has been widely used in previous studies and is considered a model system for parallel G4 structures with short loops. Including PEQ, the molecular interactions of four different small molecules with Myc2345_T23 have been described via high-resolution NMR structures, most of them just recently (Figure 7) (15,22,23). To understand the general mechanism of how small molecules recognize the parallel G4, we performed the first systematic analysis of the available complex structures involving this sequence. All reported complexes are bound by heterocyclic ligands that are positively charged at physiological pH. In addition to the new PEQ compound, the reported ligands include the asymmetric and crescent shaped quindoline derivative Qi, the symmetric and crescent shaped carbazole derivative BMVC with two arms, and the benzofuran derivative DC-34 (Figure 7A) (15,22,23). As shown for PEQ binding above (Figure 6), all four ligands recruit the first flanking base at the 5′-end (A6) or 3′-end (T23) upon binding to form a new ligand-base plane covering the external G-tetrad. This base recruitment mechanism provides a means to achieve a specific binding by engaging a flanking DNA base to lock the ligand position.

Surprisingly, although the structure, size, and shape of the four small molecules are different and the recruited G4 flanking segments are flexible, the orientation and position of the recruited residues, i.e. A6 and T23, are strikingly conserved among the different complexes (Figure 7). In all four complex structures, the position of T23 in the 3′-binding pocket remains the same as in the free G4 and the ligands replace A25 as binding partner, as described above for PEQ. In the 5′-binding pockets, A6’s orientation is reverted from the free form and conserved among all the complexes. Accordingly, the conformation of the recruited bases is inherent to the G4 sequence and less dependent on the bound ligand as revealed as a striking feature of the drug recognition of parallel G4s by our systematic structural analysis of the Myc2345_T23 ligand-complexes. This newly uncovered feature establishes an important basis for rational drug design and optimization as well as for in-situ drug screening.

## DISCUSSION

Our work provides important insights into targeting the major G4 of the *MYC* promoter by small molecules. The newly discovered quinoline-based PEQ ligand is unique and differs from most typical G4 ligands in that it is less rigid and without a large aromatic moiety. It has drug-like properties with low molecular weight, which makes it a promising lead-compound for future optimization as a potential cancer therapeutic targeting MYC G4.

The structure of the Myc2345_T23 sequence was determined in 2005 based on the rationale that the G-to-T mutation of G23 in the 3′-overhang can prevent structural polymorphism (18,33). Subsequently, the Myc2345_T23 G4 has been widely used as model structure for the major *MYC* G4 and parallel G4s in general. Numerous small molecules have been evaluated for their *MYC* G4 binding using the Myc2345_T23 G4, with a few high-resolution complex structures reported (15,22,23). However, the residue 23 in the 3′-flanking sequence was very recently shown to play a critical role for drug binding selectivity even in context of extended flanking sequences (21). Moreover, the recently reported Myc1245 structure with a 6 nt central loop bears a similar 3′-flanking sequence with the wild-type G23 and an analysis of G-register exchange showed only a marginal effect of four consecutive Gs in the last G-tract on polymorphism (26,35,36). Therefore, our new Myc2345 structure with the wild-type residue G23 in the 3′-overhang better represents the wild-type *MYC* promoter G4.

High-resolution structures provide insight into how small molecules recognize the target G4 at the atomic level and can guide structure-based rational design. PEQ specifically interacts with the MYC G4 by recruiting the 5′-flanking (A6) or 3′-flanking (G23 or T23) residues that are immediately adjacent to the G-core to form a ligand-base layer stacking on the 5′- or 3′-external G-tetrad. Such base-recruiting mechanism has been observed previously to achieve specific G4-binding (15,22,23,37,38). Interestingly, the two complex structures of PEQ with the wild-type Myc2345 and Myc2345_T23 show that the flanking segments at the two ends of the MYC G4 behave differently upon ligand binding. The 3′-binding site is rather static and the 3′-flanking residue 23 in the complexes remains in its *apo*-orientation upon the binding of PEQ, which replaces A25 in the capping base pair and forms the ligand-base plane with G23 or T23. In contrast, the 5′-flanking A6 is more dynamic and adopts an entirely different conformation in the complexes as compared to the free form, possibly due to an intrinsic dynamic nature of the 5′-flanking segment. The different dynamic nature of the 3′- and 5′-flanking segments, in combination with the lower accessibility of the 3′-tetrad (22), may explain the significant impact of the 3′-flanking residue 23 on ligand binding selectivity of MycG4 and parallel G4s, as shown in the recent large-scale G4 DNA microarray study (21). Importantly, the two complex structures demonstrate that the 3′-flanking mutant T23 residue has a notably different interface and binding conformation as compared to the wild-type G23. Thus, the complex structure with the wild-type Myc2345 should be considered as the molecular basis for future rational design of MYC G4-targeting drugs.

Intriguingly, our unprecedented systematic analysis of the four available ligand-complex structures of Myc2345_T23 uncovers similar binding pockets and in particular the well conserved orientation of the recruited bases. The same feature of ligand recognition is expected for the wild-type Myc2345, in which the orientation of the native G23 is expected to be conserved as observed in our determined PEQ complex structure. This result reveals an important mechanism of how small molecules recognize the major *MYC* G4 and more general parallel G4s with short propeller loops, i.e. small molecules recruit the flexible immediate flanking nucleotide in a conserved way specific to the recruited sequence. Such a ligand-DNA interface renders binding specificity and bears the potential for sequence selectivity in binding based on the size and chemical composition of the recruited nucleotide. This insight will not only help rationally design new or improved MYC G4-targeted drugs using our determined wild-type Myc2345 complex structure, but also enable the in-situ drug screening such as from a larger *in silico* compound library as previously tested (39).

Moreover, our results show that the interface between ligand and recruited base has great potential for the design of complementary hydrogen bonds to optimize binding selectivity. Each DNA base has a unique pattern of hydrogen bond donors and acceptors. Therefore, the development of G4 ligands can be analogous to the design of alternative base pairs, in which the functional groups of a compound are matched to the hydrogen bond pattern of the targeted base (40). However, whereas hydrogen bonds form in the 3′-capping structures of the free MYC G4s (Figure 2C and D), hydrogen bond formation with the recruited base is scarce in the reported complexes and is only observed for the 3′-binding of Qi to Myc2345_T23. This emphasizes the unused potential for the optimization of the interface between ligand and recruited base for G4 recognition.

Furthermore, electrostatic interactions are an important contribution that drive the binding to polyanionic nucleic acids (41). Two different types of electrostatic G4-interactions are observed based on the location of the positive charge within the ligand. First, a central positive charge in the aromatic moiety is often found close to the electronegative G4-inner pore, e.g. for the PEQ and Qi complex with Myc2345_T23. In this way, the ligand's charge is close to the negatively polarized carbonyl groups of the tetrad-guanines and resembles the coordinated cations within the G-core. On the other hand, positive charges at the edges or within the attached side chains are often positioned close to the negatively charged sugar-phosphate backbone in G4 grooves for favourable electrostatic interactions, as seen for the Qi, DC-34, and BMVC complexes. Therefore, the addition of positive charged side chains is a common strategy to improve G4 binding of small molecule ligands. It is noted that, as an alternative, neutral side chains may specifically interact with motifs unique for G4s, for example propeller loops (42,43).

In summary, we present the NMR solution structures of the wild-type MycG4 and its complex with the newly discovered small molecule PEQ, a specific MycG4-binder with drug-like properties. While MycG4 is the most actively pursued G4 target, the available MycG4 structure bears a mutation of a residue critical for ligand recognition. The important role of this critical residue in binding selectivity to MycG4 and parallel G4s was very recently shown by a large-scale microarray study even in the context of longer DNA sequences. Comparison of our determined structures of PEQ in complex with the wild-type and mutant MycG4 clearly shows a notable effect of this critical mutation on the drug binding interface. Therefore, our new wild-type MycG4 should replace the widely used mutant MycG4 in future studies and our complex structure provides the true molecular basis for drug recognition of the wild-type binding pocket. Furthermore, we performed an unprecedented systematic analysis of the four available complex structures involving the mutant MycG4, which can be considered a model system for parallel structures common in promoter and RNA G4s. We discovered that for recognition of parallel G4s the flexible flanking residue is recruited in a conserved way specific to the flanking sequence. This discovery establishes an important general concept for drug recognition of parallel G4s and enables rational drug design/optimization as well as in-situ drug screening using our newly determined structures.

## DATA AVAILABILITY

The coordinates for the structures of Myc2345 (accession code: 7KBV) and the 2:1 PEQ complexes with Myc2345 (accession code: 7KBW) and Myc2345_T23 (accession code: 7KBX) have been deposited in the Protein Data bank.

## Supplementary Material

gkab330_Supplemental_Files
