# Supplementary material for: Structural recognition of the MYC promoter G-quadruplex by a quinoline derivative: insights into molecular targeting of parallel G-quadruplexes
Source: Nucleic Acids Res. 2021 May 12;49(10):5905–15. doi: 10.1093/nar/gkab330 (PMC8191789; doi:10.1093/nar/gkab330)
Supplement: gkab330_Supplemental_Files [file gkab330_supplemental_files.zip › SI_NAR_final_v2.pdf]

Supporting Information

Structural Recognition of the MYC Promoter G-Quadruplex by a Quinoline Derivative: Insights into Molecular Targeting of Parallel G-Quadruplexes

Jonathan Dickerhoff, Jixun Dai, Danzhou Yang

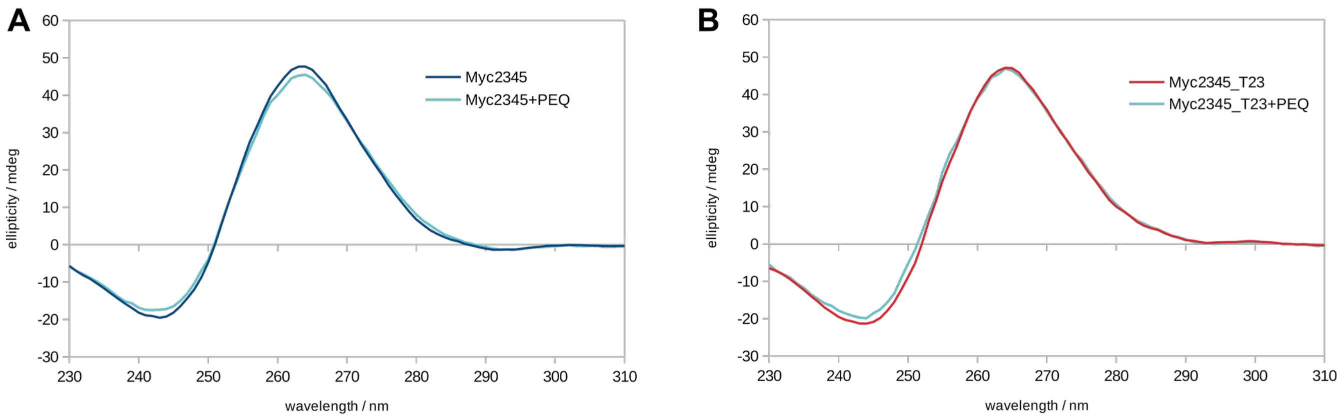

Figure S1. CD spectra of the free and PEQ bound MycG4s. Superimposed CD spectra of 5 μM (**A**) Myc2345 and (**B**) Myc2345_T23 with and without addition of PEQ (25 μM) at 25°C. Acquired at 25 °C with 100 mM K^+, pH 7.

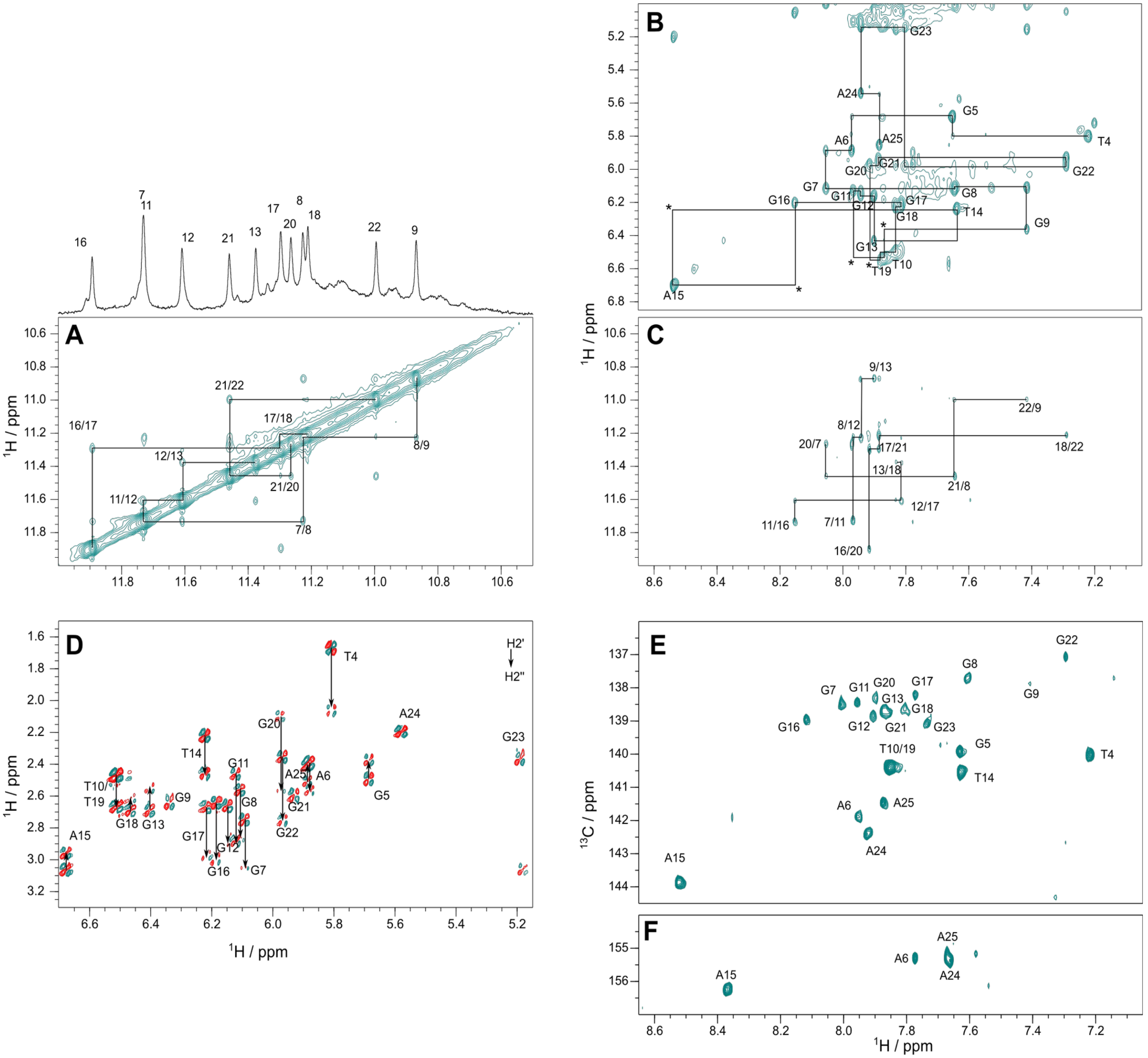

Figure S2. NMR spectra of wild-type MycG4. 2D NOE spectral regions of the Myc2345 G4 acquired at 25 °C with a mixing time of 300 ms showing (**A**) sequential imino-imino cross peaks along the parallel G-stretches, (**B**) sequential H8-H1' contacts traced by solid lines (missing NOE cross peaks are marked with an asterisk), and (**C**) guanine H8/H6–imino cross peaks showing the intra-tetrad and inter-tetrad guanine connectivities indicated by solid lines. (**D**) DQF-COSY spectral region showing the H1'-H2' and H1'-H2'' intraresidual crosspeaks for the Myc2345 quadruplex at 35 °C. The H2'/H2'' assignment is indicated by the arrow direction. Spectral regions of the 2D ^{1}H−^{13}C HSQC of Myc2345 at 25 °C showing the (**E**) H6−C6/H8−C8 peaks for all bases and the (**F**) adenine H2−C2 contacts. All spectra acquired in 10 mM K^{+} buffer, pH 7.

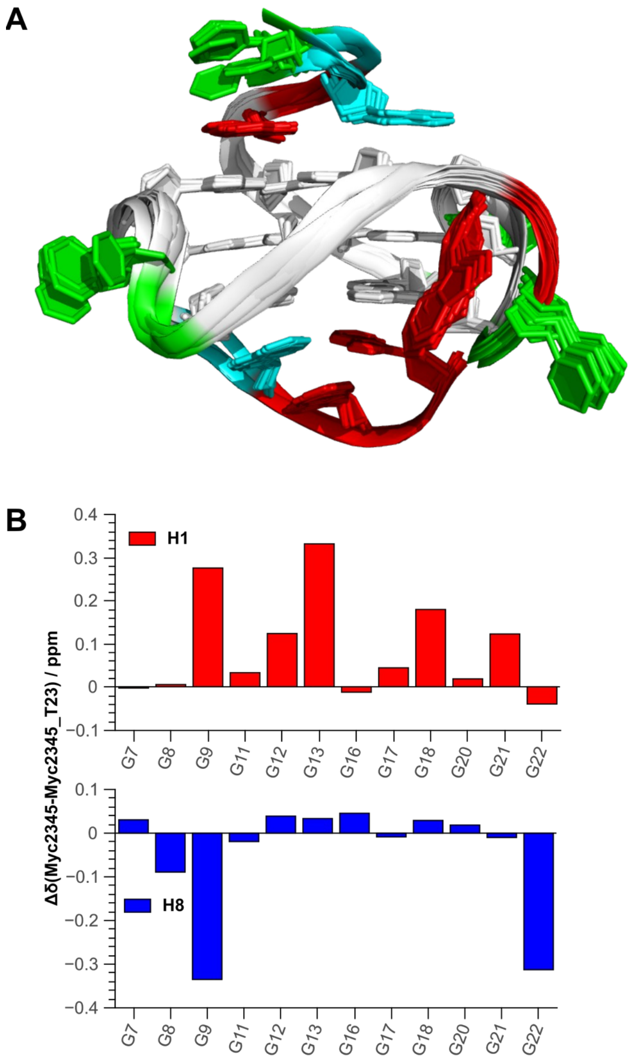

Figure S3. Structure of the wild-type MycG4 and its chemical shift difference from the mutant MycG4. (**A**) Superposition of the 10 lowest energy structures for Myc2345 (PDB: 7KBV). (**B**) NMR chemical shift differences between H1 and H8 protons within the G-core of Myc2345 and Myc2345_T23, which reflect the different positions of the capping structures.

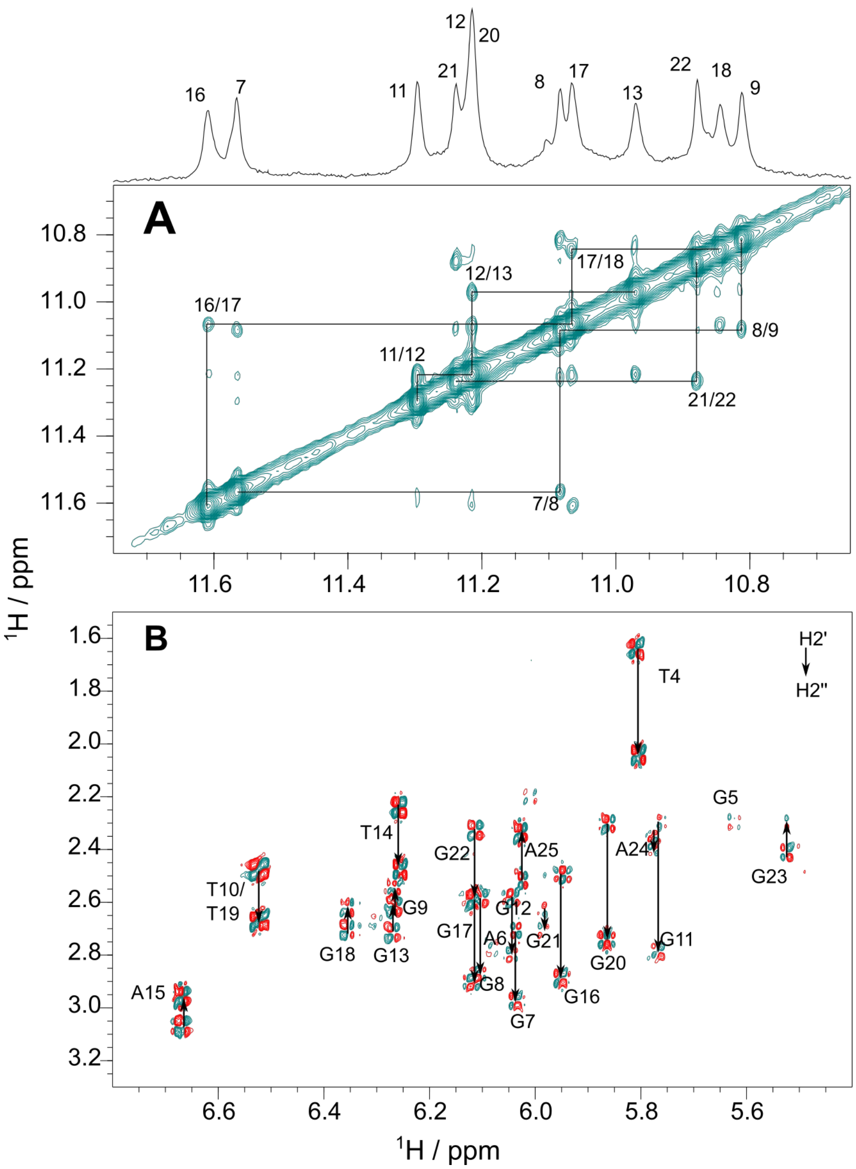

Figure S4. NMR data of wild-type MycG4-PEQ complex**.** (**A**) 2D NOE spectral region of the 2:1 PEQ-Myc2345 complex with PEQ acquired at 25 °C with a mixing time of 300 ms that shows sequential imino-imino cross peaks along the parallel G-stretches. (**B**) DQF-COSY spectral region showing the H1'-H2' and H1'-H2'' intraresidual crosspeaks for the 2:1 PEQ-Myc2345 complex at 35 °C. The H2'/H2'' assignment is indicated by the arrow direction. All spectra acquired in 10 mM K^+ buffer, pH 7.

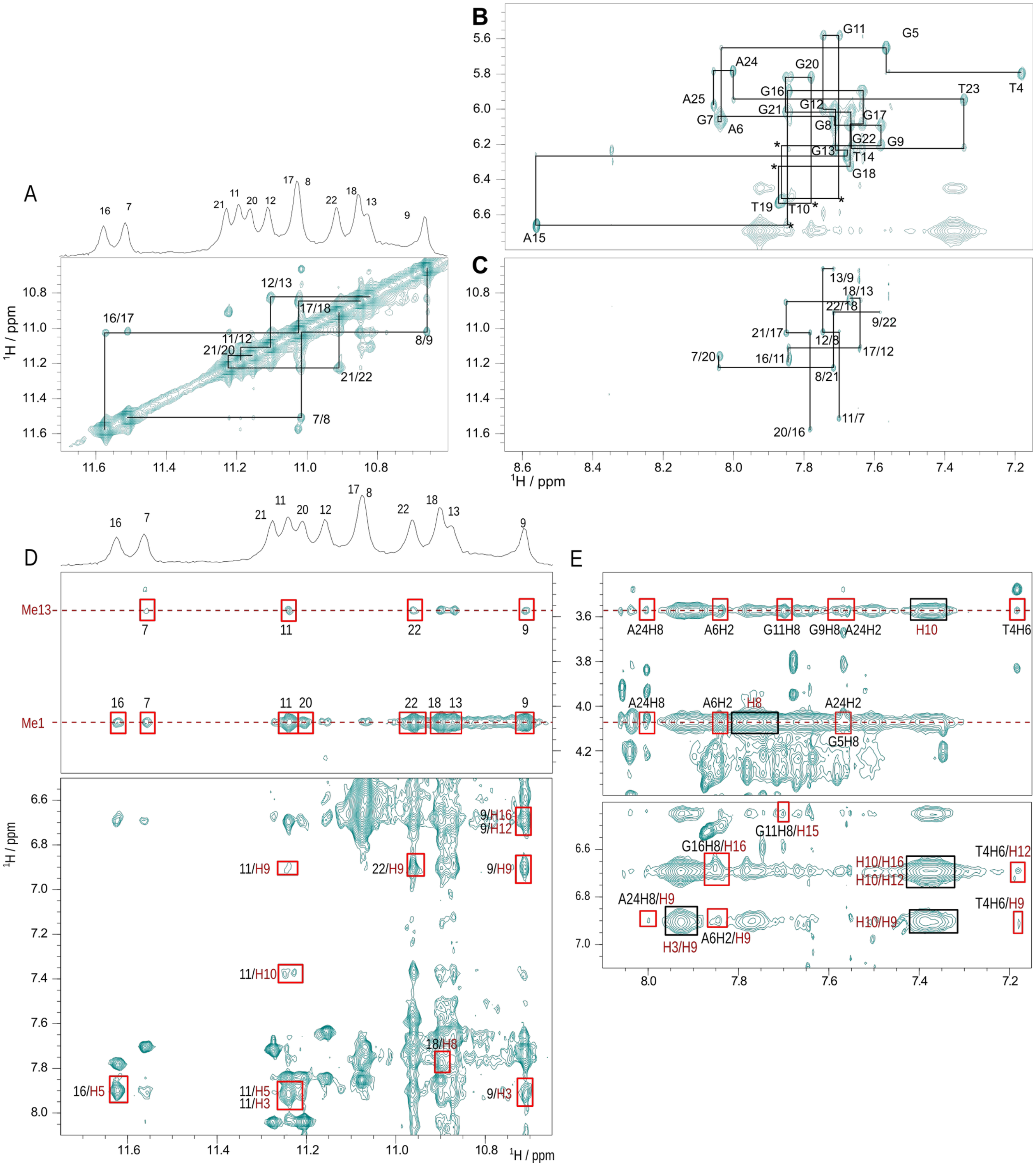

Figure S5. NMR assignment and inter-molecular NOE (red) of the mutant MycG4-PEQ complex**.** 2D NOE spectral regions of the 2:1 PEQ-Myc2345_T23 complex with PEQ. (**A**) Sequential imino-imino cross peaks along the parallel G-stretches. (**B**) Sequential H8-H1' contacts traced by solid lines. Missing NOE cross peaks are marked with an asterisk. (**C**) Guanine H8/H6–imino cross peaks showing the intra-tetrad and inter-tetrad guanine connectivities indicated by solid lines. Intermolecular contacts between PEQ and (**D**) G-core imino protons and (**E**) H2/H6/H8 aromatic protons. PEQ protons are labeled in red. Strong intramolecular NOE cross peaks of PEQ are indicated by a black box. Spectrum measured at 20 °C with 10 mM K^+, pH 6.5 and 3:1 drug-DNA ratio. NOESY mixing time is 300 ms.

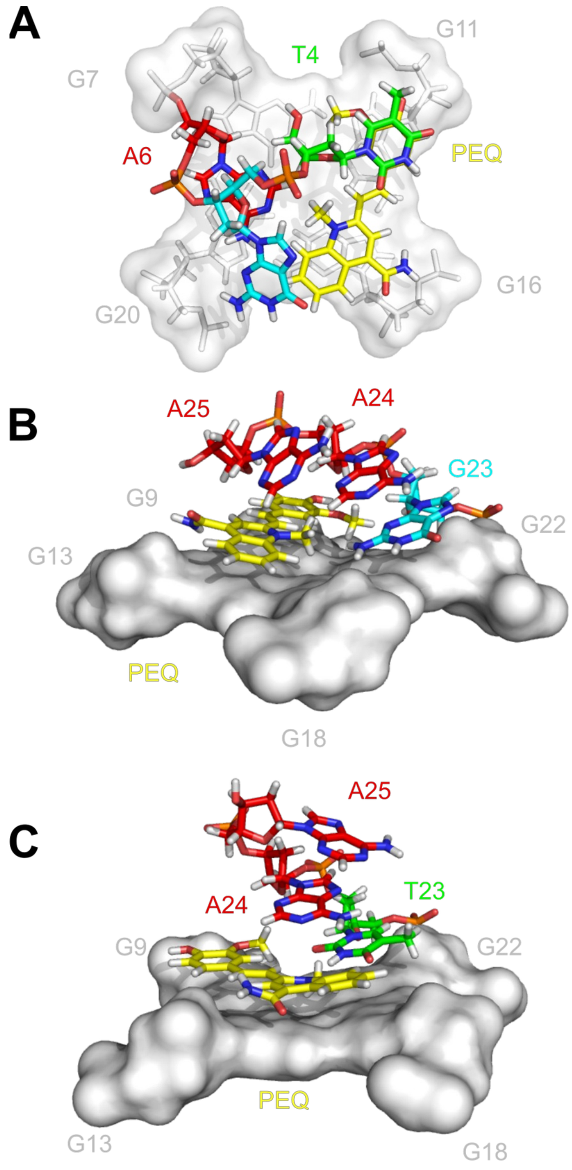

Figure S6. PEQ interactions with the flanking sequences**.** Top and side view of the 5'- and 3'-binding of PEQ including all flanking residues, respectively. (**A**) 5'-binding of PEQ to the Myc2345. 3'-binding of PEQ to (**B**) Myc2345 (PDB: 7KBW) and (**C**) Myc2345_T23. (PDB: 7KBX)

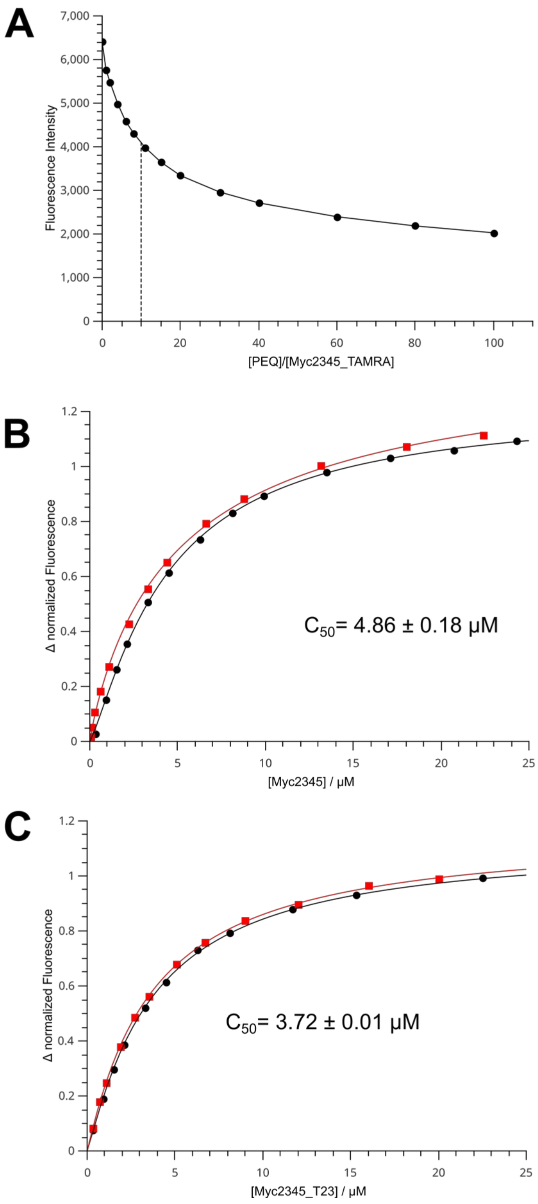

Figure S7. Relative binding affinity of PEQ to the wild-type and mutant MycG4**.** (**A**) Initial titration of PEQ to 3'-TAMRA-labeled Myc2345_T23 to determine the best ligand-DNA ratio for the competition experiments. A 10:1 ratio of PEQ to the TAMRA-labeled Myc2345_T23 was chosen to obtain significant TAMRA quenching while limiting the excess of PEQ in solution. (**B**) Myc2345 and (**C**) Myc2345_T23 were titrated to the 10:1 mixture of PEQ and 3'-TAMRA labeled Myc2345_T23 in duplicate (black and red) to determine the C_{50} values. The concentration of the labeled MYC2345_T23 was 100 nM in 100 mM K^+ buffer, pH 7. λ_{ex}= 555 nm, λ_{em}= 579 nm

Table S1. CD melting temperatures of 5 μM Myc2345 and Myc2345_T23 with and without PEQ (25 μM). Measured in duplicate with 0.6 mM K^+, pH 7.

Sample	T_m / °C	ΔT_m(complex-free) / °C
Myc2345	57.5 ± 0.2	
Myc2345_T23	54.6 ± 0.3	
Myc2345+PEQ	77.6 ± 0.5	20.1 ± 0.6
Myc2345_T23+PEQ	75.7 ± 1.3	21.1 ± 1.3

Table S2. ^{1}H and C8/C6 ^{13}C chemical shifts (ppm) of the Myc2345 quadruplex.[a]

	imino	H8/H6	H2/Me	H1’	H2’/H2’’	H3’	H4’	H5’/H5’’[b]	C6/C8
T4	---	7.22	1.67	5.80	1.63/2.05	4.44	3.82	3.48/3.48	139.9
G5	---	7.65	---	5.68	2.54/2.40	4.84	4.12	3.74/3.83	139.7
A6	---	7.97	7.78	5.89	2.39/2.57	4.84	4.01	3.63/3.85	141.6
G7	11.73	8.05	---	6.11	2.79/3.07	5.00	4.47	4.03/4.12	138.21
G8	11.23	7.64	---	6.11	2.59/2.86	5.01	4.52	4.28/4.31	137.64
G9	10.87	7.42	---	6.36	2.70/2.67	5.16	4.58	4.30/4.36	137.53
T10	---	7.87	2.00	6.53	2.48/2.68	5.11	4.61	4.30/4.36	140.24
G11	11.73	7.97	---	6.13	2.46/2.90	5.11	4.47	4.26/4.34	138.26
G12	11.61	7.94	--	6.16	2.69/2.88	5.09	4.48	4.23/4.28	138.71
G13	11.38	7.90	---	6.43	2.72/2.56	5.04	4.49	4.28/4.31	138.51
T14	---	7.64	1.94	6.24	2.21/2.46	4.72	3.88	3.72/3.75	140.33
A15	---	8.54	8.38	6.70	3.09/2.98	5.20	4.61	4.22/4.31	143.73
G16	11.89	8.15	---	6.20	2.68/3.03	5.06	4.49	4.18/4.29	138.82
G17	11.29	7.81	---	6.23	2.70/2.99	5.04	4.56	4.24/4.29	138.05
G18	11.21	7.83	---	6.49	2.71/2.64	5.15	4.64	4.31/4.42	138.47
T19	---	7.87	2.00	6.54	2.49/2.69	5.13	4.63	4.31/4.37	140.25
G20	11.27	7.92	---	5.97	2.35/2.76	5.09	4.46	4.28/4.34	138.1
G21	11.46	7.89	---	5.93	2.61/2.62	5.05	4.49	4.17/4.24	138.66
G22	11.00	7.29	---	5.98	2.09/2.56	4.90	4.47	4.13/4.22	136.77
G23	---	7.80	---	5.14	2.44/2.36	4.84	4.38	4.11/4.18	138.89
A24	---	7.94	7.66	5.54	2.20/2.19	4.58	2.94	3.58/3.65	142.19
A25	---	7.88	7.66	5.85	2.49/2.43	4.44	4.10	3.69/3.89	141.05

[a]In 90% H_2O/10% D_2O, 10 mM potassium phosphate buffer, pH 7.0 at 25 °C; n.d. = not determined. [b]No stereospecific assignments.

Table S3. Sequential and long-range NOE interactions of the Myc2345 quadruplex involving the 5'-terminal residues T4-A6.[a]

T4		H1'	H2'	H2''	H3'	H4'	H5'	H5''	H1	Me
G5	H3'			a						
	H8	w	w	m	m	w				
A6	H2'									v
	H2''									w
	H2									w
	H8	v	w	w						
G20	H8									v

G5		H1'	H2'	H2''	H3'	H4'	H5'	H5''	H1	H8
T4	H1'									w
	H2'									w
	H2''				a					m
	H3'									m
	H4'									w
A6	H8	w	m	s	m	w	w	w		m
G7	H1	w	w	m						
G11	H1	w								w
G16	H1									v

A6		H1'	H2'	H2''	H3'	H4'	H5'	H5''	H1	H2	H8
T4	H1'										v
	H2'										w
	H2''										w
	Me		v	w						w	
G5	H1'										w
	H2'										m
	H2''										s
	H3'										m
	H4'										w
	H5'										w
	H5''										w
	H8										m
G7	H1	v									
	H8	m	w	m	w	s	w	w			
G20	H1'									m	
	H1	m								v	m

[a]Cross peak intensities are classified as strong (s), medium (m), weak (w), very weak (v), or ambiguous (a) in case of overlapped resonances.

Table S4. Sequential and long-range NOE interactions of the Myc2345 quadruplex involving the 3'-terminal residues G23-A25.[a]

**G23**		**H1'**	**H2'**	**H2''**	**H3'**	**H4'**	**H5'**	**H5''**	**H1**		**H8**
**G22**	**H1'**						m	m			w
	**H2'**										m
	**H2''**										m
	**H3'**										v
	**H8**										m
**A24**	**H8**	m	m	m	w						
**A25**	**H2**	w									

**A24**		**H1'**	**H2'**	**H2''**	**H3'**	**H4'**	**H5'**	**H5''**	**H1**	**H2**	**H8**
**G23**	**H1'**										m
	**H2'**										m
	**H2''**										m
	**H3'**										w
**A25**	**H2'**									m	
	**H2''**									w	
	**H2**					w	w	w			
	**H8**	m	a	a	w	m					

**A25**		**H1'**	**H2'**	**H2''**	**H3'**	**H4'**	**H5'**	**H5''**	**H1**	**H2**	**H8**
**G9**	**H1'**	m									
	**H2'**									m	
	**H2''**									m	
	**H1**	v	w	w							w
	**H8**									m	v
**G23**	**H1'**									w	
**A24**	**H1'**										m
	**H2'**										a
	**H2''**										a
	**H3'**										w
	**H4'**									w	m
	**H5'**									w	
	**H5''**									w	
	**H2**		m	w							

[a]Cross peak intensities are classified as strong (s), medium (m), weak (w), very weak (v), or ambiguous (a) in case of overlapped resonances.

Table S5. ^{1}H and C8/C6 ^{13}C chemical shifts (ppm) of the 2:1 PEQ-Myc2345 complex.[a]

	imino	H8/H6	H2/Me	H1'	H2'/H2''	H3'	H4'	H5'/H5''[b]	C6/C8
T4	---	7.20	1.60	5.80	1.62/2.04	4.44	3.83	3.48/3.48	139.89
G5	---	7.53	---	5.62	2.30/2.31	4.71	3.98	3.62/3.74	139.56
A6	---	8.02	7.88	6.07	2.80/2.77	4.96	4.36	3.98/4.05	142.50
G7	11.56	8.05	---	6.04	2.73/2.99	5.02	4.50	4.16/4.21	138.96
G8	11.09	7.68	---	6.11	2.60/2.87	5.01	4.55	4.30/4.32	137.96
G9	10.81	7.58	---	6.27	2.62/2.56	5.13	4.57	4.30/4.34	138.00
T10	---	7.88	2.01	6.53	2.48/2.67	5.10	4.58	4.25/4.34	140.23
G11	11.30	7.79	---	5.73	2.28/2.79	5.04	4.36	4.25/4.31	138.53
G12	11.22	7.82	–-	6.05	2.59/2.80	5.05	4.44	4.18/4.25	138.73
G13	10.97	7.78	---	6.28	2.73/2.61	5.04	4.47	4.25/4.28	138.38
T14	---	7.68	1.96	6.27	2.24/2.48	4.74	3.94	3.78/3.82	140.42
A15	---	8.57	8.38	6.69	3.09/2.97	5.20	4.61	4.21/4.31	143.71
G16	11.61	7.88	---	5.95	2.51/290	5.01	4.50	n.d.	138.65
G17	11.07	7.67	---	6.13	2.61/2.91	5.02	4.55	4.23/4.26	138.00
G18	10.85	7.73	---	6.37	2.72/2.64	5.16	4.64	4.31/4.38	138.22
T19	---	7.89	2.01	6.54	2.49/2.68	5.10	4.61	4.29/4.37	140.28
G20	11.22	7.81	---	5.87	2.30/2.75	5.06	4.44	4.28/4.34	138.18
G21	11.24	7.86	---	5.99	2.65/2.71	5.09	4.53	4.19/4.27	138.71
G22	10.88	7.51	---	6.12	2.33/2.58	4.99	4.45	4.19/4.27	137.43
G23	---	7.65	---	5.48	2.42/2.31	4.84	4.31	4.13/4.14	139.92
A24	---	7.99	7.60	5.74	2.34/2.40	4.79	3.93	3.85/3.92	142.02
A25	---	8.06	7.68	6.01	2.51/2.33	4.57	4.04	3.92/3.97	142.13

[a]In 90% H_2O/10% D_2O, 10 mM potassium phosphate buffer, pH 7.0, 3:1 PEQ-Myc2345 ratio, at 25 °C; n.d. = not determined. [b]No stereospecific assignments.

Table S6. ^{1}H chemical shifts (ppm) of PEQ in the 2:1 PEQ-Myc2345 complex in 90% H_2O/10% D_2O, 10 mM potassium phosphate buffer, pH 7.0, 3:1 PEQ-Myc2345 ratio at 25 °C

	Me1	H3	H5	H6	H7	H8	H9	H10	H12	Me13	H15	H16
PEQ	4.12	7.95	7.87	7.46	7.57	7.73	6.96	7.38	6.72	3.58	6.62	6.66

Table S7. ¹H chemical shifts (ppm) of the 2:1 PEQ-Myc2345_T23 complex.[a]

	imino	H8/H6	H2/Me	H1'	H2'/H2''	H3'	H4'	H5'/H5''[b]
T4	---	7.18	1.56	5.80	1.63/2.06	4.46	3.83	3.48/3.48
G5	---	7.57	---	5.65	2.33/2.33	4.76	4.03	3.67/3.79
A6	---	8.04	7.84	6.07	2.87/2.78	4.99	4.40	4.06/4.10
G7	11.51	8.04	---	6.04	2.73/2.99	5.03	4.52	4.19/4.22
G8	11.02	7.71	---	6.09	2.61/2.82	5.02	4.53	4.21/4.30
G9	10.66	7.58	---	6.20	2.58/2.50	5.11	4.54	4.28/4.32
T10	---	7.87	2.00	6.52	2.46/2.65	5.07	4.56	4.22/4.32
G11	11.19	7.70	---	5.58	2.19/2.72	5.00	4.29	n.d.
G12	11.11	7.74	---	6.00	2.53/2.75	5.02	4.40	4.13/4.22
G13	10.83	7.72	---	6.23	2.72/2.57	5.03	4.54	4.23/4.26
T14	---	7.68	1.95	6.26	2.22/2.47	4.73	3.95	3.78/3.81
A15	---	8.56	8.35	6.66	3.07/2.95	5.18	4.59	4.20/4.29
G16	11.57	7.84	---	5.90	2.47/2.87	4.99	4.49	4.24/4.30
G17	11.02	7.64	---	6.08	2.57/2.86	5.01	4.52	4.22/4.24
G18	10.85	7.67	---	6.32	2.67/2.58	5.14	4.57	4.35/4.53
T19	---	7.87		6.53	2.48/2.67	5.09	4.59	4.26/4.35
G20	11.16	7.78	---	5.82	2.27/2.73	5.04	4.14	4.26/4.32
G21	11.22	7.85	---	6.02	2.64/2.73	5.08	4.53	4.18/4.27
G22	10.91	7.66	---	6.22	2.56/2.69	5.03	4.53	4.23/4.30
T23	---	7.35	1.78	5.94	1.89/2.17	4.80	4.23	4.11/4.18
A24	---	8.00	7.55	5.78	2.30/2.42	4.78	4.06	3.84/3.93
A25	---	8.06	2.00	5.98	2.47/2.28	4.55	3.96	3.90/3.93

[a]In 90% H_2O/10% D_2O, 10 mM potassium phosphate buffer, pH 6.5, 3:1 PEQ-Myc2345_T23 ratio at 20 °C; n.d. = not determined. [b]No stereospecific assignments.

Table S8. ¹H chemical shifts (ppm) of PEQ in the 2:1 PEQ-Myc2345_T23 complex in 90% H_2O/10% D_2O, 10 mM potassium phosphate buffer, pH 6.5, 3:1 PEQ-Myc2345_T23 ratio at 20 °C

	Me1	H3	H5	H6	H7	H8	H9	H10	H12	Me13	H15	H16
PEQ	4.07	7.92	7.89	7.50	7.65	7.77	6.90	7.37	6.69	3.57	6.45	6.69

Table S9. Sequential and long-range intramolecular NOE interactions of Myc2345 and Myc2345_T23 in their 2:1 complex with PEQ involving the 5'-terminal residues T4-A6.[a]

		**Myc2345**								**Myc2345_T23**							
**T4**		**H1'**	**H2'**	**H2''**	**H3'**	**H4'**	**H5'**	**H5''**	**H8**	**H1'**	**H2'**	**H2''**	**H3'**	**H4'**	**H5'**	**H5''**	**H8**
**G5**	**H5'**	a		w						a		w					
	**H5''**	a		w						a		w					
	**H8**	w	w	w	w	w				v	w	w	w	w			
**A6**	**H8**						a	a							a	a	

**G5**		**H1'**	**H2'**	**H2''**	**H3'**	**H4'**	**H5'**	**H5''**	**H8**	**H1'**	**H2'**	**H2''**	**H3'**	**H4'**	**H5'**	**H5''**	**H8**
**T4**	**H1'**						a	a	w						a	a	v
	**H2'**								w								w
	**H2''**						w	w	w						w	w	w
	**H3'**								w								w
	**H4'**								w								w
**A6**	**H5'**	w								w							
	**H5''**	w								w							
	**H2**	v				w			w	v							
	**H8**	w	w	w	w		v	v		w	a	a	w	m			

**A6**		**H1'**	**H2'**	**H2''**	**H3'**	**H4'**	**H5'**	**H5''**	**H2**	**H8**	**H1'**	**H2'**	**H2''**	**H3'**	**H4'**	**H5'**	**H5''**	**H2**	**H8**
**T4**	**H5'**									a									a
	**H5''**									a									a
**G5**	**H1'**						w	w	v	w						w	w	v	w
	**H2'**									w									a
	**H2''**									w									a
	**H3'**									w									w
	**H4'**								w										m
	**H5'**									v									
	**H5''**									v									
	**H8**								w										
**G7**	**H5'**	a									a								
	**H5''**	a									a								
	**H1**								w									w	
	**H8**	m	m	m	w	w					m	m	m	w	w				
**G20**	**H1**	w							w	w	w							w	w

[a]Cross peak intensities are classified as strong (s), medium (m), weak (w), very weak (v), or ambiguous (a) in case of overlapped resonances.

Table S10. Sequential and long-range intramolecular NOE interactions of Myc2345 and Myc2345_T23 in their 2:1 complex with PEQ involving the 3’-terminal residues G23/T23-A25.[a]

Myc2345

G23		H1'	H2'	H2''	H3'	H4'	H5'	H5''	H2	H8
G22	H1'				w		m	m		w
	H2'									w
	H2''						a	a		m
	H3'									w
A24	H5'	m								v
	H5''	m								
	H8	m	m	m	w	v				w
A25	H2	v								

A24		H1'	H2'	H2''	H3'	H4'	H5'	H5''	H2	H8
G23	H1'						m	m		m
	H2'									m
	H2''									m
	H3'									w
	H4'									v
	H8						v			w
A25	H1'								w	
	H5'	w								
	H5''	w								
	H8	m	w	m	w		w	w		

A25		H1'	H2'	H2''	H3'	H4'	H5'	H5''	H2	H8
G9	H1’	w								
G23	H1’								v	
A24	H1'						w	w		m
	H2'									w
	H2''									m
	H3'									w
	H5'									w
	H5''									w
	H2	w								

Myc2345_T23

T23		H1'	H2'	H2''	H3'	H4'	H5'	H5''	H2	Me	H6
G18	H1									w	
G22	H1'						a	a		w	m
	H2'									m	m
	H2''									m	m
	H3'									w	w
	H8									m	m
A24	H5'	a									
	H5''	a									
	H8	w	m	m	a					v	w

A24		H1'	H2'	H2''	H3'	H4'	H5'	H5''	H2	H8
T23	H1'						a	a		w
	H2'									m
	H2''									m
	H3'									a
	Me									v
	H6									w
A25	H1'								w	
	H5'	m								
	H5''	m								
	H8	w	m	m	w	w	w	w		

A25		H1'	H2'	H2''	H3'	H4'	H5'	H5''	H2	H8
G9	H1’									
G23	H1’									
A24	H1'						m	m		w
	H2'									m
	H2''									m
	H3'									w
	H4’									w
	H5'									w
	H5''									w
	H2	w								

[a]Cross peak intensities are classified as strong (s), medium (m), weak (w), very weak (v), or ambiguous (a) in case of overlapped resonances.

Table S11. Intermolecular NOE interactions of Myc2345 or Myc2345_T23 with PEQ in the 2:1 complex that define the 5'-binding.[a]

Myc2345

		Me1	H3	H5	H6	H8	H9	H10	H12	H15	H16	Me13
**T4**	**H6**						w		w			m
	**Me**											w
**G5**	**H8**	m										
**A6**	**H2**	s					w					m
**G7**	**H1**	w										v
**G11**	**H1**	m	w				w	w				w
	**H8**									m		m
**G16**	**H1**	w		w								
	**H8**										m	
**G20**	**H1**	w										

Myc2345_T23

		Me1	H3	H5	H6	H8	H9	H10	H12	H15	H16	Me13
**T4**	**H6**	w					w		w			m
	**Me**											w
**G5**	**H8**	m										
**A6**	**H2**	s					w					m
**G7**	**H1**	w										v
**G11**	**H1**	m	w	v			w	w				w
	**H8**									m		m
**G16**	**H1**	w		w								
	**H8**										m	
**G20**	**H1**	w										

[a]Cross peak intensities are classified as strong (s), medium (m), weak (w), or very weak (v).

Table S12. Intermolecular NOE interactions of Myc2345 or Myc2345_T23 with PEQ in the 2:1 complex that define the 3'-binding.[a]

Myc2345

		Me1	H3	H5	H6	H8	H9	H12	H15	H16	Me13
**G9**	**H1**	m	w					w		w	v
	**H8**										m
	**H1'**										w
	**H2''**										w
**G13**	**H1**	m									
**G18**	**H1**	m				w					
**G22**	**H1**	m					w				w
**G23**	**H8**										w
	**H2''**										w
	**H4'**										m
**A24**	**H2**	m									w
	**H8**	w					w				w
	**H1'**										

Myc2345_T23

		Me1	H3	H5	H6	H8	H9	H12	H15	H16	Me13
**G9**	**H1**	m	w				w	w		w	w
	**H8**										m
	**H1'**										
	**H2''**										
**G13**	**H1**	m									
**G18**	**H1**	m				w					
**G22**	**H1**	m					w				w
**T23**	**H1'**										m
	**H4'**										w
**A24**	**H2**	m									m
	**H8**	w					w				w

[a]Cross peak intensities are classified as strong (s), medium (m), weak (w), or very weak (v).
